# Crystal structure of poly[(*N*,*N*-di­methyl­acetamide-κ*O*)(μ_4_-5-methyl­isophthalato-κ^5^ *O*:*O*,*O*′:*O*′′:*O*′′′)manganese(II)]

**DOI:** 10.1107/S2056989014025626

**Published:** 2015-01-01

**Authors:** Lan Jin, Li-Li Zha, San Gao, Shi-Yao Yang, Rong-Bin Huang

**Affiliations:** a Department of Chemistry, College of Chemistry and Chemical Engineering, Xiamen University, Xiamen 361005, People’s Republic of China

**Keywords:** crystal structure, manganese(II) coordination polymer, pcu structure, *N*,*N*-di­methyl­acetamide, 5-methyl­isophthalate

## Abstract

The title compound, poly[(*N*,*N*-di­methyl­acetamide-κ*O*)(μ_4_-5-methyl­isophthalato-κ^5^ *O*,*O*′:*O*′,*O*′′:*O*′′)manganese(II)], [Mn(C_9_H_6_O_4_)(C_3_H_7_NO)]_*n*_, was obtained from a mixture containing MnCl_2_·4H_2_O and 5-methyl­isophthalic acid in *N*,*N*-di­methyl­acetamide solution. The Mn^2+^ ion is coordinated by five O atoms from four bridging 5-methyl­isophthalate ligands and one O atom from one *N*,*N*-di­methyl­acetamide ligand, defining a considerably distorted coordination polyhedron with one very long Mn—O bond of 2.623 (2) Å. The Mn^2+^ ions are joined by carboxyl­ate groups, forming rod-shaped secondary building units along the *a* axis. The rods are further connected by 5-methyl­isophthalate ligands to form the pcu (primitive cubic net) structure.

## Related literature   

For the structures of coordination polymers comprising first-row transition metal ions and benzene di­carboxyl­ates, see: Deng *et al.* (2013 ▸); Jin *et al.* (2012 ▸); Li *et al.* (2010 ▸); Yang *et al.* (2013 ▸); Zhou *et al.* (2009 ▸). For the nomenclature for metal-organic frameworks, see: Rosi *et al.* (2005 ▸);. A very closely related crystal structure, poly[(di­methyl­formamide)(5-meth­oxy­benzene-1,3-di­carboxyl­ato)manganese(II)], was reported recently (Huang, 2013 ▸). The author described the structure in a PtS (cooperite) topology according to a different analytical approach (Carlucci *et al.*, 2003 ▸; Hill *et al.*, 2005 ▸).
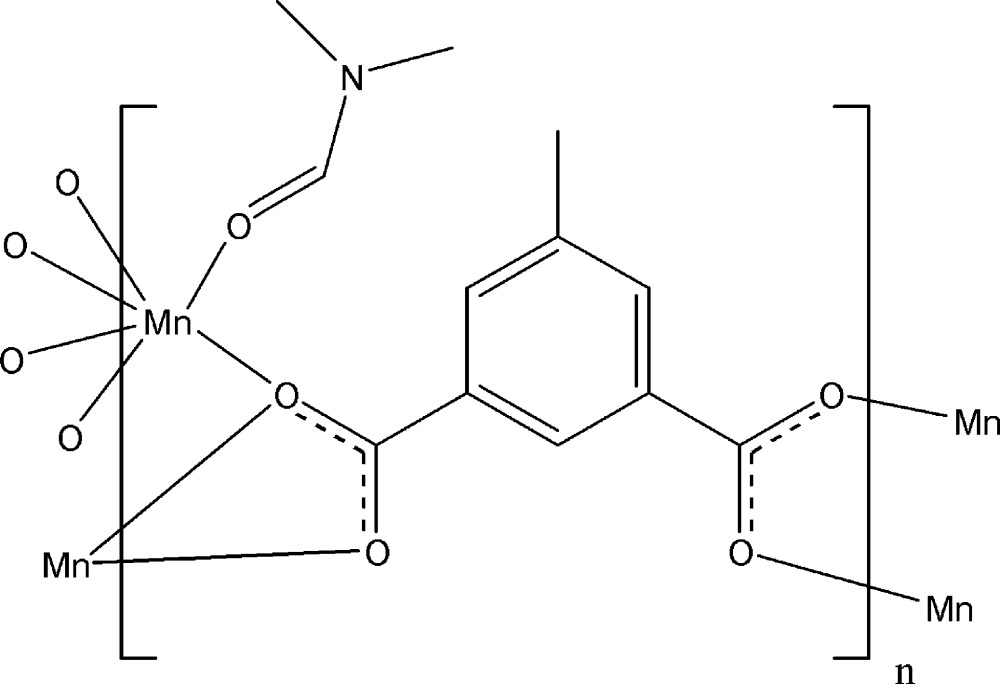


## Experimental   

### Crystal data  

[Mn(C_9_H_6_O_4_)(C_3_H_7_NO)]
*M* *_r_* = 306.17
Orthorhombic, 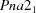

*a* = 7.281 (5) Å
*b* = 15.148 (11) Å
*c* = 10.903 (8) Å
*V* = 1202.5 (15) Å^3^
*Z* = 4
Mo *K*α radiation
μ = 1.11 mm^−1^
*T* = 200 K
0.15 × 0.10 × 0.10 mm

### Data collection  

Bruker APEX area-detector diffractometer
Absorption correction: multi-scan (*SADABS*; Sheldrick, 2004 ▸) *T*_min_ = 0.851, *T*_max_ = 0.897
10159 measured reflections
2874 independent reflections
2768 reflections with *I* > 2σ(*I*)
*R*_int_ = 0.025

### Refinement  

*R*[*F* ^2^ > 2σ(*F* ^2^)] = 0.024
*wR*(*F* ^2^) = 0.059
*S* = 1.07
2874 reflections
175 parameters
1 restraint
H-atom parameters constrained
Δρ_max_ = 0.26 e Å^−3^
Δρ_min_ = −0.29 e Å^−3^
Absolute structure: Flack (1983 ▸), 0 Friedel pairs
Absolute structure parameter: 0.025 (14)

### 

Data collection: *SMART* (Bruker, 2002 ▸); cell refinement: *SAINT* (Bruker, 2002 ▸); data reduction: *SAINT*; program(s) used to solve structure: *SHELXTL* (Sheldrick, 2008 ▸); program(s) used to refine structure: *SHELXTL*; molecular graphics: *DIAMOND* (Brandenburg, 2007 ▸); software used to prepare material for publication: *SHELXTL*.

## Supplementary Material

Crystal structure: contains datablock(s) I, AE2. DOI: 10.1107/S2056989014025626/fk2084sup1.cif

Structure factors: contains datablock(s) I. DOI: 10.1107/S2056989014025626/fk2084Isup2.hkl

**Fig. 1.**
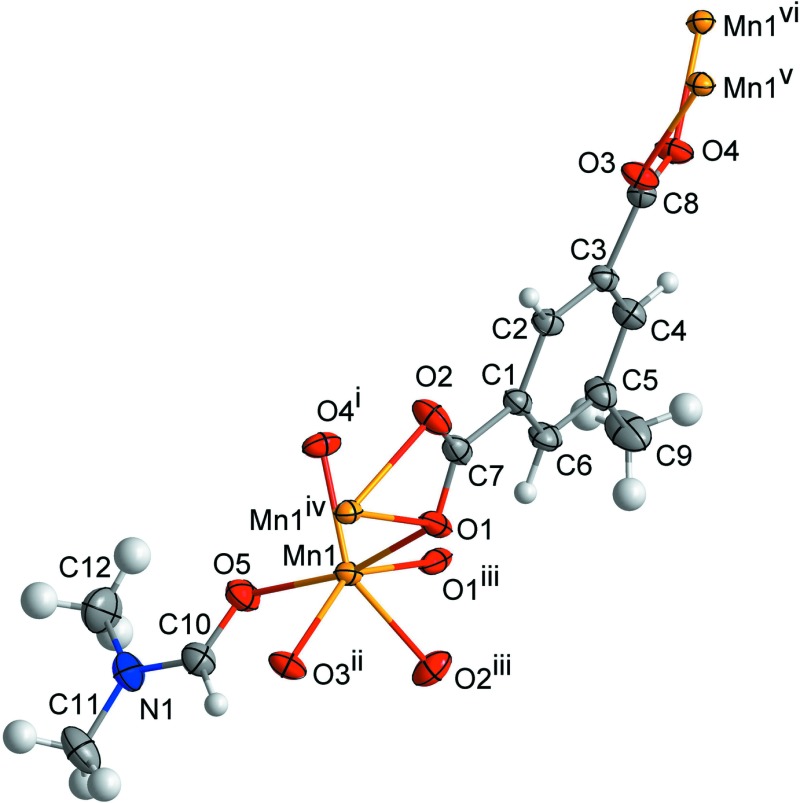
Coordination modes in (I). Anisotropic displacement ellipsoids are drawn at the 50% probability level. Symmetry codes: i -x, -y + 2, z + 1/2; ii -x + 1/2, y - 1/2, z + 1/2; iii x - 1/2, -y + 3/2, z; iv x + 1/2, -y + 3/2, z; v -x + 1/2, y + 1/2, z - 1/2; vi -x, -y + 2, z - 1/2.

**Fig. 2.**
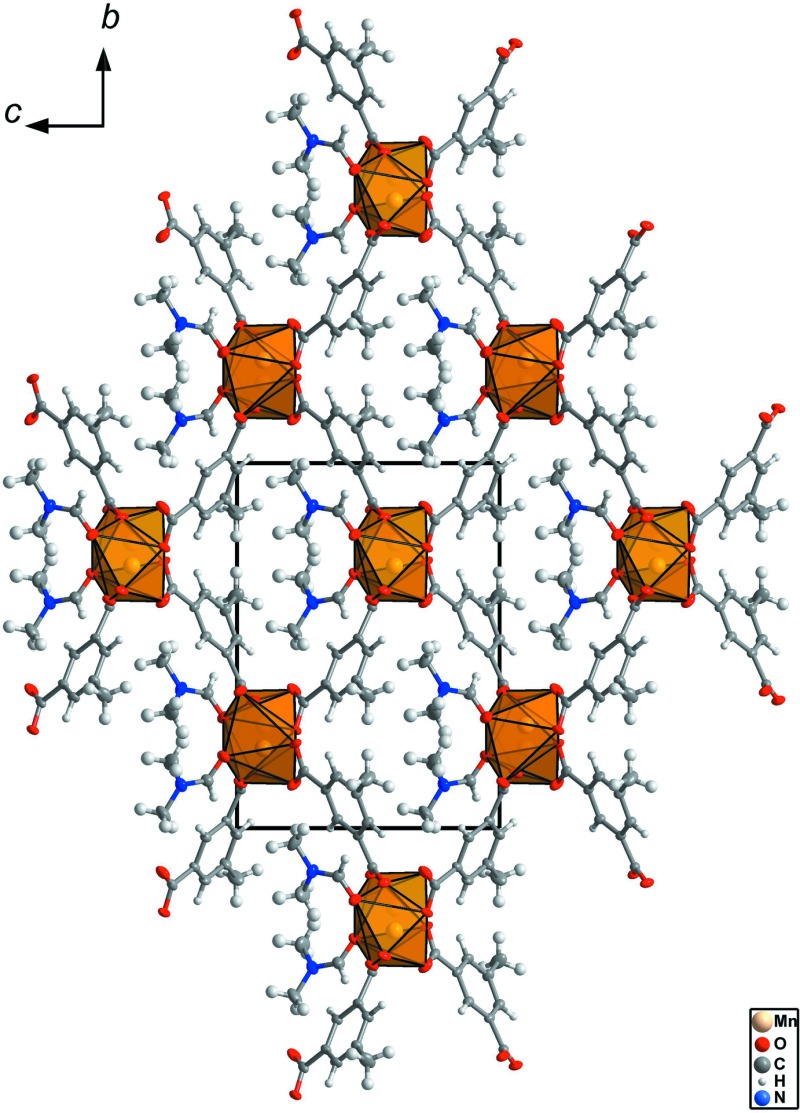
The packing of (I), viewed down the a axis, showing MnO6 in polyhedra.

Click here for additional data file.x y z x y z x y z x y z x y z x y z . DOI: 10.1107/S2056989014025626/fk2084fig1.tif
Coordination modes in (I). Anisotropic displacement ellipsoids are drawn at the 50% probability level. Symmetry codes: i −*x*, −*y* + 2, *z* + 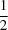; ii −*x* + 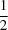, *y* − 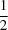, *z* + 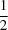; iii *x* − 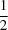, −*y* + 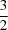, *z*; iv *x* + 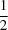, −*y* + 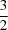, *z*; v −*x* + 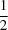, *y* + 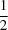, *z* − 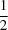; vi −*x*, −*y* + 2, *z* − 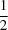.

a 6 . DOI: 10.1107/S2056989014025626/fk2084fig2.tif
The packing of (I), viewed down the *a* axis, showing MnO_6_ in polyhedra.

CCDC reference: 1035658

Additional supporting information:  crystallographic information; 3D view; checkCIF report

## Figures and Tables

**Table:** 

Mn1O4^i^	2.0609(19)
Mn1O3^ii^	2.0855(15)
Mn1O1	2.0885(16)
Mn1O5	2.1342(18)
Mn1O2^iii^	2.1378(16)

**Table:** 

O4^i^Mn1O3^ii^	131.59(6)
O4^i^Mn1O1	83.59(6)
O3^ii^Mn1O1	98.77(7)
O4^i^Mn1O5	83.95(6)
O3^ii^Mn1O5	84.88(7)
O1Mn1O5	166.04(5)
O4^i^Mn1O2^iii^	135.70(6)
O3^ii^Mn1O2^iii^	92.23(7)
O1Mn1O2^iii^	97.51(7)
O5Mn1O2^iii^	95.80(7)

Symmetry codes: (i) 
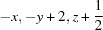
; (ii) 
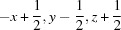
; (iii) 
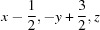
.

## supplementary crystallographic information

### S1. Structural commentary

We have reported the structures of dozens of coordination polymers comprising first-row transition metal ions and benzene di­carboxyl­ates (Deng *et al.*, 2013; Jin *et al.*, 2012; Li *et al.* 2010; Yang *et al.*, 2013; Zhou *et al.*, 2009). We found that metal ion is the most important factor that influences the structure of coordination polymer.

The title compound, [Mn(C_9_H_6_O_4_)(C_3_H_7_O)]_n_, (I) was obtained with the same method reported in our previous paper (Yang *et al.*, 2013). The structure feature of I is quite similar to those manganese analogs reported in that paper. The Mn^2+^ ions are joined by carboxyl groups to form rod-shaped secondary building units (SBUs) along the *a* axis. Each rod is further connected to four adjacent rods by 5-methyl­isophthalates to form the rod packing type 2 pcu (primitive cubic net) structure according to the nomenclature for metal-organic frameworks (Rosi *et al.*, 2005). A very closely related molecular structure, poly[(di­methyl­formamide)(5-meth­oxy­benzene-1,3-di­carboxyl­ato)manganese(II)], was reported recently (Huang, 2013). The author described the structure in PtS (cooperite) topology according to a different analysis approach (Carlucci *et al.*, 2003; Hill *et al.*, 2005).

### S2. Synthesis and crystallization

A mixture containing MnCl_2_.4H_2_O (0.039 g, 0.20 mmol) and 5-methyl­isophthalic acid (H_2_mip, 0.036 g, 0.20 mmol) in 10 mL *N*,*N*-di­methyl­acetamide (DMF) was heated at 100 °C for 5000 min. Colourless block crystals were generated (0.025 g, 41%).

### S3. Refinement

Crystal data, data collection and structure refinement details are summarized in Table 1. H atoms bonded to C atoms were positioned geometrically and refined using a riding model (including free rotation about the C—C bond), with C—H = 0.95–0.99 Å and with *U*_iso_(H) = 1.2 (1.5 for methyl groups) times *U*_eq_(C).

### Crystal data

**Table:**

[Mn(C_9_H_6_O_4_)(C_3_H_7_NO)]	*F*(000) = 628
*M**_r_* = 306.17	*D*_x_ = 1.691 Mg m^−^^3^
Orthorhombic, *P**n**a*2_1_	Mo *K*α radiation, λ = 0.71073 Å
Hall symbol: P 2c -2n	Cell parameters from 6647 reflections
*a* = 7.281 (5) Å	θ = 2.3–28.7°
*b* = 15.148 (11) Å	µ = 1.11 mm^−^^1^
*c* = 10.903 (8) Å	*T* = 200 K
*V* = 1202.5 (15) Å^3^	Rod, colorless
*Z* = 4	0.15 × 0.10 × 0.10 mm

### Data collection

**Table:**

Bruker APEX area-detector diffractometer	2874 independent reflections
Radiation source: fine-focus sealed tube	2768 reflections with *I* > 2σ(*I*)
Graphite monochromator	*R*_int_ = 0.025
φ and ω scan	θ_max_ = 29.0°, θ_min_ = 2.3°
Absorption correction: multi-scan (*SADABS*; Sheldrick, 2004)	*h* = −9→9
*T*_min_ = 0.851, *T*_max_ = 0.897	*k* = −19→19
10159 measured reflections	*l* = −13→14

### Refinement

**Table:**

Refinement on *F*^2^	Secondary atom site location: difference Fourier map
Least-squares matrix: full	Hydrogen site location: inferred from neighbouring sites
*R*[*F*^2^ > 2σ(*F*^2^)] = 0.024	H-atom parameters constrained
*wR*(*F*^2^) = 0.059	*w* = 1/[σ^2^(*F*_o_^2^) + (0.0316*P*)^2^ + 0.1079*P*] where *P* = (*F*_o_^2^ + 2*F*_c_^2^)/3
*S* = 1.07	(Δ/σ)_max_ = 0.001
2874 reflections	Δρ_max_ = 0.26 e Å^−^^3^
175 parameters	Δρ_min_ = −0.29 e Å^−^^3^
1 restraint	Absolute structure: Flack (1983), 0 Friedel pairs
Primary atom site location: structure-invariant direct methods	Absolute structure parameter: 0.025 (14)

### Special details

**Table:**

Geometry. All e.s.d.'s (except the e.s.d. in the dihedral angle between two l.s. planes) are estimated using the full covariance matrix. The cell e.s.d.'s are taken into account individually in the estimation of e.s.d.'s in distances, angles and torsion angles; correlations between e.s.d.'s in cell parameters are only used when they are defined by crystal symmetry. An approximate (isotropic) treatment of cell e.s.d.'s is used for estimating e.s.d.'s involving l.s. planes.
Refinement. Refinement of *F*^2^ against ALL reflections. The weighted *R*-factor *wR* and goodness of fit *S* are based on *F*^2^, conventional *R*-factors *R* are based on *F*, with *F* set to zero for negative *F*^2^. The threshold expression of *F*^2^ > σ(*F*^2^) is used only for calculating *R*-factors(gt) *etc*. and is not relevant to the choice of reflections for refinement. *R*-factors based on *F*^2^ are statistically about twice as large as those based on *F*, and *R*- factors based on ALL data will be even larger.

### Fractional atomic coordinates and isotropic or equivalent isotropic displacement parameters (Å^2^)

**Table:**

	*x*	*y*	*z*	*U*_iso_*/*U*_eq_	
Mn1	0.02668 (3)	0.718473 (13)	0.40075 (4)	0.01733 (7)	
O1	0.21890 (19)	0.76885 (7)	0.27699 (13)	0.0264 (3)	
O2	0.40794 (19)	0.87833 (8)	0.28213 (14)	0.0320 (3)	
O3	0.30783 (16)	1.12474 (7)	−0.01315 (13)	0.0244 (3)	
O4	0.02059 (16)	1.15017 (8)	−0.06146 (13)	0.0254 (3)	
O5	−0.15683 (19)	0.69631 (9)	0.55025 (14)	0.0301 (3)	
N1	−0.2451 (2)	0.61847 (11)	0.71232 (16)	0.0323 (4)	
C1	0.1457 (2)	0.89377 (11)	0.16063 (16)	0.0210 (3)	
C2	0.1976 (2)	0.97512 (10)	0.11805 (18)	0.0208 (3)	
H2A	0.3104	1.0003	0.1442	0.025*	
C3	0.0878 (2)	1.02002 (10)	0.03817 (16)	0.0196 (3)	
C4	−0.0754 (2)	0.98383 (11)	0.00205 (17)	0.0237 (3)	
H4A	−0.1529	1.0157	−0.0523	0.028*	
C5	−0.1290 (3)	0.90257 (12)	0.04273 (18)	0.0269 (4)	
C6	−0.0157 (3)	0.85839 (12)	0.12133 (19)	0.0261 (4)	
H6A	−0.0505	0.8014	0.1494	0.031*	
C7	0.2643 (2)	0.84485 (11)	0.24625 (17)	0.0236 (4)	
C8	0.1438 (2)	1.10570 (10)	−0.01549 (17)	0.0197 (3)	
C9	−0.3076 (3)	0.86426 (15)	0.0039 (3)	0.0448 (6)	
H9A	−0.2980	0.7997	0.0020	0.067*	
H9B	−0.3392	0.8861	−0.0780	0.067*	
H9C	−0.4034	0.8817	0.0623	0.067*	
C10	−0.1507 (3)	0.63087 (12)	0.61313 (19)	0.0282 (4)	
H10A	−0.0704	0.5849	0.5880	0.034*	
C11	−0.2297 (3)	0.53842 (16)	0.7799 (2)	0.0459 (6)	
H11A	−0.1283	0.5032	0.7469	0.069*	
H11B	−0.3446	0.5050	0.7731	0.069*	
H11C	−0.2057	0.5520	0.8664	0.069*	
C12	−0.3792 (4)	0.68114 (17)	0.7505 (3)	0.0545 (7)	
H12A	−0.3596	0.7369	0.7069	0.082*	
H12B	−0.3680	0.6911	0.8390	0.082*	
H12C	−0.5022	0.6586	0.7319	0.082*	

### Atomic displacement parameters (Å^2^)

**Table:**

	*U*^11^	*U*^22^	*U*^33^	*U*^12^	*U*^13^	*U*^23^
Mn1	0.01845 (11)	0.01338 (10)	0.02015 (12)	0.00142 (7)	0.00145 (16)	0.00084 (12)
O1	0.0343 (7)	0.0170 (5)	0.0279 (7)	0.0074 (5)	0.0119 (6)	0.0068 (5)
O2	0.0262 (7)	0.0330 (7)	0.0368 (8)	−0.0002 (6)	−0.0060 (6)	0.0145 (6)
O3	0.0188 (6)	0.0207 (5)	0.0336 (7)	−0.0019 (4)	0.0012 (5)	0.0087 (5)
O4	0.0232 (6)	0.0172 (6)	0.0359 (8)	0.0006 (4)	−0.0074 (5)	0.0059 (5)
O5	0.0333 (7)	0.0266 (6)	0.0305 (8)	0.0002 (6)	0.0079 (6)	0.0069 (6)
N1	0.0328 (9)	0.0366 (9)	0.0274 (10)	−0.0036 (7)	0.0051 (7)	0.0065 (7)
C1	0.0227 (8)	0.0191 (7)	0.0211 (8)	0.0021 (6)	0.0043 (6)	0.0043 (6)
C2	0.0192 (8)	0.0193 (7)	0.0240 (8)	0.0002 (6)	0.0026 (6)	0.0021 (6)
C3	0.0202 (8)	0.0175 (7)	0.0212 (8)	0.0010 (6)	0.0047 (7)	0.0019 (6)
C4	0.0217 (8)	0.0230 (8)	0.0264 (9)	0.0006 (6)	0.0005 (7)	0.0053 (7)
C5	0.0267 (9)	0.0274 (9)	0.0267 (9)	−0.0067 (7)	−0.0001 (7)	0.0044 (7)
C6	0.0295 (9)	0.0201 (8)	0.0286 (10)	−0.0051 (6)	0.0048 (7)	0.0040 (7)
C7	0.0262 (8)	0.0235 (8)	0.0213 (9)	0.0070 (7)	0.0060 (7)	0.0051 (7)
C8	0.0223 (8)	0.0168 (7)	0.0199 (8)	0.0008 (6)	0.0000 (6)	0.0005 (6)
C9	0.0371 (12)	0.0452 (12)	0.0521 (14)	−0.0188 (9)	−0.0127 (10)	0.0132 (11)
C10	0.0253 (9)	0.0291 (9)	0.0303 (10)	−0.0008 (7)	0.0022 (8)	0.0026 (8)
C11	0.0445 (13)	0.0526 (13)	0.0407 (14)	−0.0074 (10)	0.0018 (11)	0.0222 (11)
C12	0.0674 (17)	0.0524 (14)	0.0437 (15)	0.0092 (13)	0.0238 (13)	−0.0037 (12)

### Geometric parameters (Å, º)

**Table:**

Mn1—O4^i^	2.0609 (19)	C2—C3	1.364 (2)
Mn1—O3^ii^	2.0855 (15)	C2—H2A	0.9500
Mn1—O1	2.0885 (16)	C3—C4	1.367 (2)
Mn1—O5	2.1342 (18)	C3—C8	1.481 (2)
Mn1—O2^iii^	2.1378 (16)	C4—C5	1.365 (2)
O1—C7	1.244 (2)	C4—H4A	0.9500
O2—C7	1.226 (2)	C5—C6	1.365 (3)
O2—Mn1^iv^	2.1378 (16)	C5—C9	1.485 (3)
O3—C8	1.229 (2)	C6—H6A	0.9500
O3—Mn1^v^	2.0855 (15)	C9—H9A	0.9800
O4—C8	1.228 (2)	C9—H9B	0.9800
O4—Mn1^vi^	2.0609 (18)	C9—H9C	0.9800
O5—C10	1.206 (2)	C10—H10A	0.9500
N1—C10	1.295 (3)	C11—H11A	0.9800
N1—C11	1.424 (3)	C11—H11B	0.9800
N1—C12	1.424 (3)	C11—H11C	0.9800
C1—C6	1.361 (3)	C12—H12A	0.9800
C1—C2	1.370 (2)	C12—H12B	0.9800
C1—C7	1.472 (2)	C12—H12C	0.9800
			
O4^i^—Mn1—O3^ii^	131.59 (6)	C4—C5—C9	120.65 (19)
O4^i^—Mn1—O1	83.59 (6)	C1—C6—C5	121.77 (17)
O3^ii^—Mn1—O1	98.77 (7)	C1—C6—H6A	119.1
O4^i^—Mn1—O5	83.95 (6)	C5—C6—H6A	119.1
O3^ii^—Mn1—O5	84.88 (7)	O2—C7—O1	121.56 (17)
O1—Mn1—O5	166.04 (5)	O2—C7—C1	119.62 (16)
O4^i^—Mn1—O2^iii^	135.70 (6)	O1—C7—C1	118.76 (17)
O3^ii^—Mn1—O2^iii^	92.23 (7)	O4—C8—O3	126.10 (17)
O1—Mn1—O2^iii^	97.51 (7)	O4—C8—C3	116.16 (16)
O5—Mn1—O2^iii^	95.80 (7)	O3—C8—C3	117.70 (15)
C7—O1—Mn1	133.66 (12)	C5—C9—H9A	109.5
C7—O2—Mn1^iv^	104.72 (11)	C5—C9—H9B	109.5
C8—O3—Mn1^v^	135.39 (11)	H9A—C9—H9B	109.5
C8—O4—Mn1^vi^	137.13 (12)	C5—C9—H9C	109.5
C10—O5—Mn1	122.70 (13)	H9A—C9—H9C	109.5
C10—N1—C11	120.94 (19)	H9B—C9—H9C	109.5
C10—N1—C12	120.75 (18)	O5—C10—N1	125.04 (18)
C11—N1—C12	118.1 (2)	O5—C10—H10A	117.5
C6—C1—C2	119.04 (17)	N1—C10—H10A	117.5
C6—C1—C7	120.53 (16)	N1—C11—H11A	109.5
C2—C1—C7	120.42 (17)	N1—C11—H11B	109.5
C3—C2—C1	120.22 (17)	H11A—C11—H11B	109.5
C3—C2—H2A	119.9	N1—C11—H11C	109.5
C1—C2—H2A	119.9	H11A—C11—H11C	109.5
C2—C3—C4	119.58 (15)	H11B—C11—H11C	109.5
C2—C3—C8	121.87 (16)	N1—C12—H12A	109.5
C4—C3—C8	118.50 (15)	N1—C12—H12B	109.5
C5—C4—C3	121.09 (17)	H12A—C12—H12B	109.5
C5—C4—H4A	119.5	N1—C12—H12C	109.5
C3—C4—H4A	119.5	H12A—C12—H12C	109.5
C6—C5—C4	118.27 (17)	H12B—C12—H12C	109.5
C6—C5—C9	121.07 (18)		
			
O4^i^—Mn1—O1—C7	−2.37 (18)	Mn1^iv^—O2—C7—O1	−2.1 (2)
O3^ii^—Mn1—O1—C7	−133.56 (19)	Mn1^iv^—O2—C7—C1	−179.51 (13)
O5—Mn1—O1—C7	−29.3 (4)	Mn1—O1—C7—O2	104.6 (2)
O2^iii^—Mn1—O1—C7	132.99 (19)	Mn1—O1—C7—C1	−78.0 (2)
O4^i^—Mn1—O5—C10	−152.61 (17)	C6—C1—C7—O2	−178.90 (18)
O3^ii^—Mn1—O5—C10	−19.79 (16)	C2—C1—C7—O2	2.0 (3)
O1—Mn1—O5—C10	−125.7 (2)	C6—C1—C7—O1	3.7 (3)
O2^iii^—Mn1—O5—C10	71.94 (17)	C2—C1—C7—O1	−175.45 (17)
C6—C1—C2—C3	0.5 (3)	Mn1^vi^—O4—C8—O3	−26.5 (3)
C7—C1—C2—C3	179.64 (16)	Mn1^vi^—O4—C8—C3	155.59 (13)
C1—C2—C3—C4	0.8 (3)	Mn1^v^—O3—C8—O4	−12.1 (3)
C1—C2—C3—C8	−176.42 (16)	Mn1^v^—O3—C8—C3	165.74 (13)
C2—C3—C4—C5	−1.3 (3)	C2—C3—C8—O4	−163.04 (17)
C8—C3—C4—C5	176.03 (17)	C4—C3—C8—O4	19.7 (2)
C3—C4—C5—C6	0.4 (3)	C2—C3—C8—O3	18.9 (2)
C3—C4—C5—C9	179.3 (2)	C4—C3—C8—O3	−158.37 (17)
C2—C1—C6—C5	−1.4 (3)	Mn1—O5—C10—N1	172.24 (15)
C7—C1—C6—C5	179.47 (18)	C11—N1—C10—O5	179.4 (2)
C4—C5—C6—C1	0.9 (3)	C12—N1—C10—O5	5.1 (3)
C9—C5—C6—C1	−178.0 (2)		

Symmetry codes: (i) −*x*, −*y*+2, *z*+1/2; (ii) −*x*+1/2, *y*−1/2, *z*+1/2; (iii) *x*−1/2, −*y*+3/2, *z*; (iv) *x*+1/2, −*y*+3/2, *z*; (v) −*x*+1/2, *y*+1/2, *z*−1/2; (vi) −*x*, −*y*+2, *z*−1/2.
