# Supplementary figures and images for: Crystal structure of poly[(N,N-di­methyl­acetamide-κO)(μ4-5-methyl­isophthalato-κ5 O:O,O′:O′′:O′′′)manganese(II)]
Source: Acta Crystallogr E Crystallogr Commun. 2015 Jan 1;71(Pt 1):m1–2. doi: 10.1107/S2056989014025626 (PMC4331860; doi:10.1107/S2056989014025626)

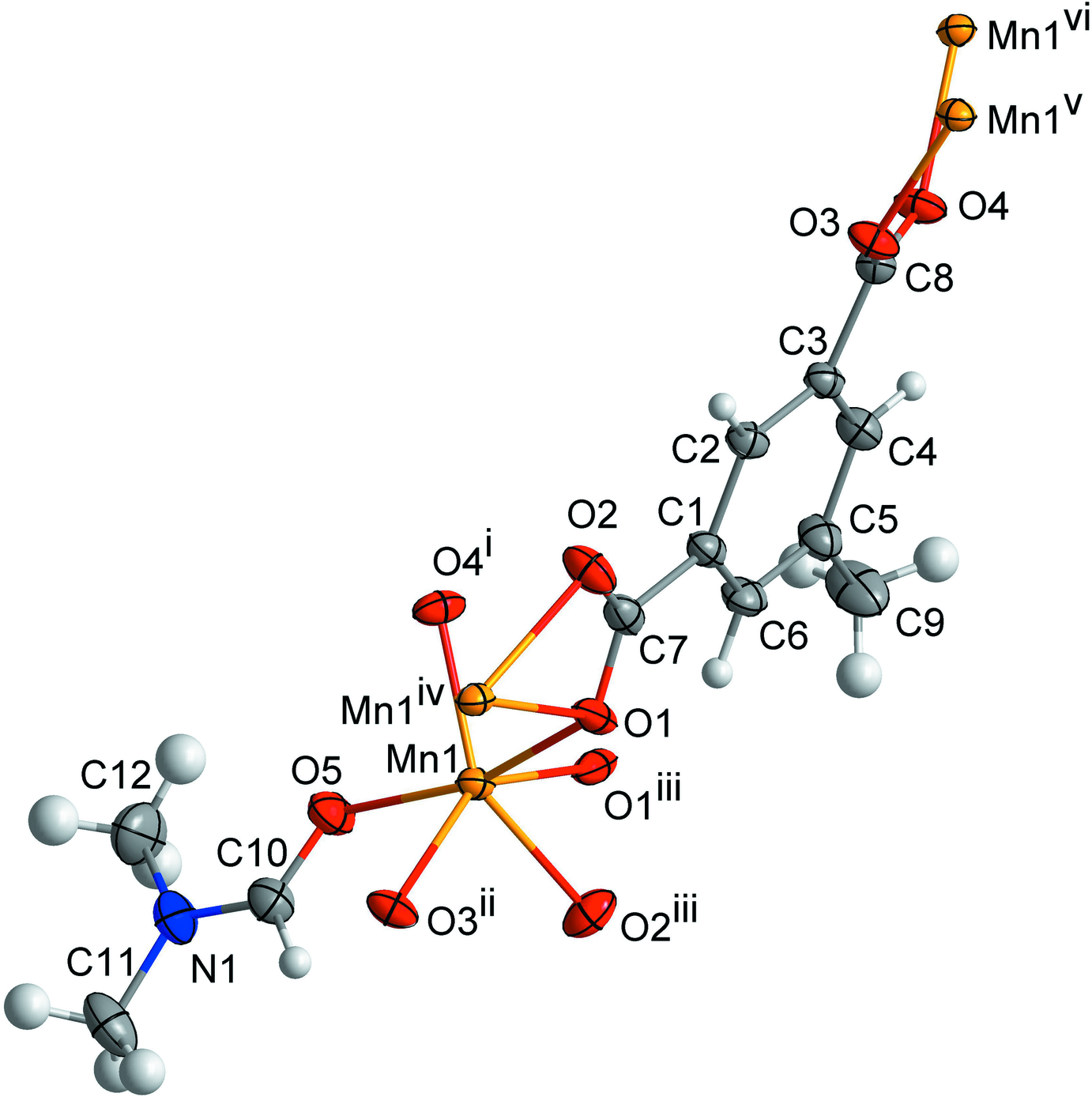

Supplement: Supplementary file 3 [file e-71-000m1-fig1.tif]

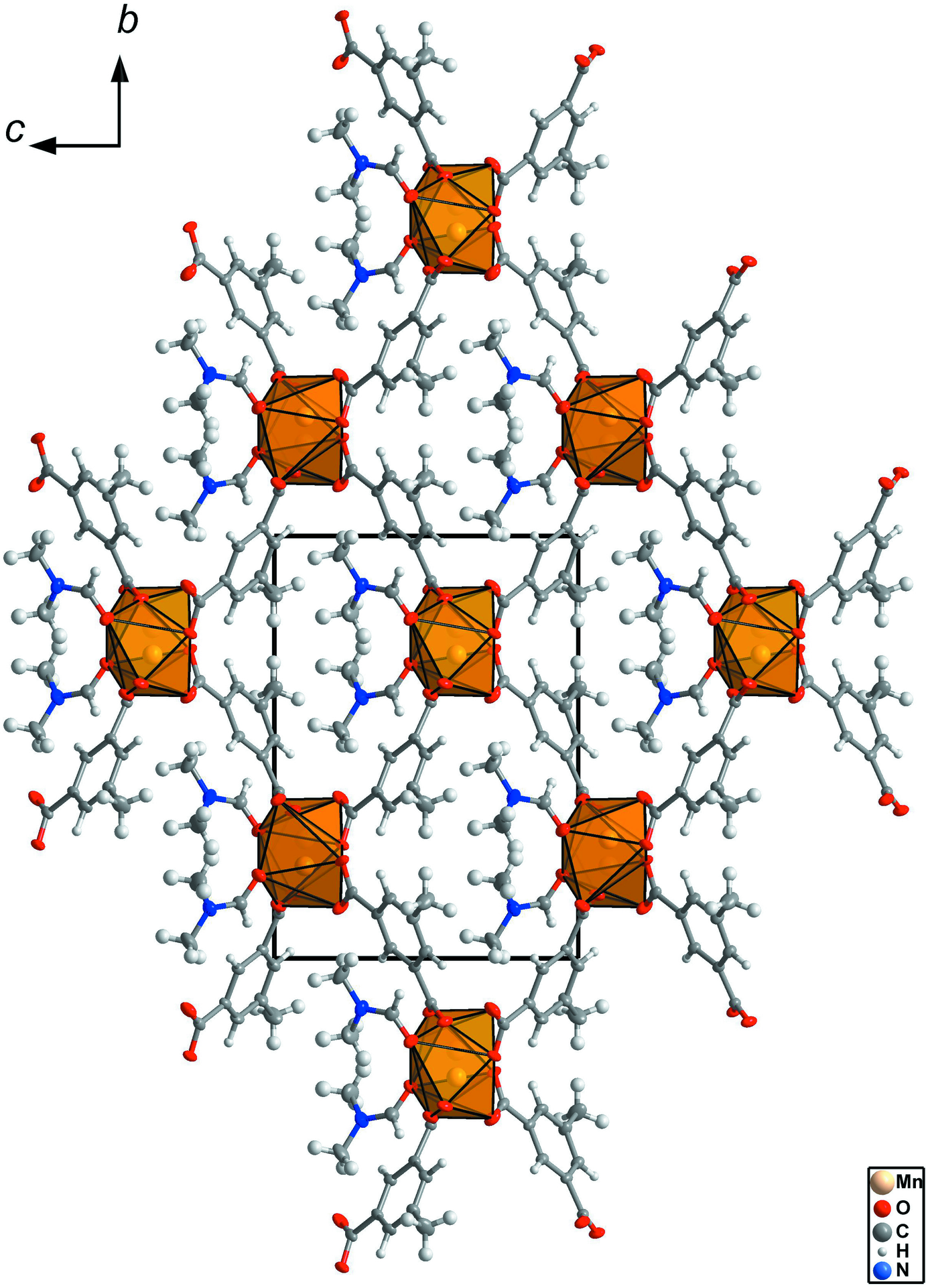

Supplement: Supplementary file 4 [file e-71-000m1-fig2.tif]
